# Review of Alzheimer's disease scales: is there a need for a new multi-domain scale for therapy evaluation in medical practice?

**DOI:** 10.1186/alzrt48

**Published:** 2010-08-26

**Authors:** Philippe Robert, Steven Ferris, Serge Gauthier, Ralf Ihl, Bengt Winblad, Frank Tennigkeit

**Affiliations:** 1 Memory Center, CHU - University of Nice Sophia Antipolis, Hôpital de Cimiez, 4 av Victoria, 06000 Nice, France; 2 Alzheimer's Disease Center, New York University Langone Medical Center, 550 First Avenue, New York, NY 10016, USA; 3 Alzheimer's Disease Research Unit, McGill Center for Studies in Aging, 6825 LaSalle Boulevard, Montreal, QC, Canada; 4 Department of Geriatric Psychiatry and Psychotherapy, Alexian Hospital, Oberdiessemer Str. 136, 47805 Krefeld, Germany; 5 Alzheimer Disease Research Center, Karolinska Institutet, Novum 5th Floor, SE-14186 Stockholm, Sweden; 6 Merz Pharmaceuticals, Eckenheimer Landstr. 100, 60318 Frankfurt am Main, Germany

## Abstract

**Introduction:**

The present review of Alzheimer's disease (AD) rating scales aims to outline the need for a new rating scale to be used in routine clinical practice for long-term medical care of AD patients. An *ideal *scale would be: 1) practical, easy and quick to administer for an experienced clinician; 2) validated for AD; 3) multi-domain: covering the AD-relevant areas of cognition, activities of daily living, behavior, communication/social interaction, and quality of life; 4) applicable to all AD severity stages; 5) able to monitor disease progression; and 6) sensitive to measure therapy effects.

**Methods:**

The National Library of Medicines' MEDLINE database was searched for the years 1981 to September 2008, using a set of keywords aiming to select instruments which cover at least some of the requirements for an ideal practical AD scale for therapy evaluation. Measures for AD staging and screening tests were not considered for review.

**Results:**

Of 1,902 articles resulting from the literature search, 68 relevant AD scales were identified. Most of them were scales that predominantly measure the severity of major dysfunctions in particular AD domains. Only five scales met some of the requirements for a practical multi-domain AD scale, but did not possess all required characteristics.

**Conclusions:**

Despite the multitude of AD scales for various purposes, there remains a need for a new multi-domain and easy to administer AD scale for assessment of disease progression and response to therapy in daily medical practice.

## Introduction

Alzheimer's disease (AD), together with other forms of dementia, represents a major challenge for health care systems with aging populations. AD is associated with neurodegenerative changes which compromise not only cognitive functioning but also lead to a decline in functional abilities and induce a spectrum of psychological or behavioral symptoms [1]. Many efforts are currently undertaken to investigate AD pathology and develop appropriate treatment strategies. These strategies focus on long-term preservation of cognitive and functional abilities or slowing down disease progression along with reducing behavioral symptoms and maintaining the patient's quality of life [2,3]. As long as there is no treatment leading to reversal or stopping of disease progression, an amelioration of the disease symptoms, which may delay institutionalization, as well as a reduction of caregiver burden and costs, are realistic treatment goals today.

Rating scales are essential tools for AD diagnosis, staging, assessment and careful monitoring of AD symptoms as well as for evaluation of treatment effects. For decades most AD assessments were predominantly focused on cognition, which is the *lead *symptom in AD. Nevertheless, it has been realized that the symptoms more relevant to a patient's quality of life, caregiver burden and institutionalization are functional and behavioral symptoms [4]. In the 1990 s, the development of new measures was requested by regulatory authorities to demonstrate clinically relevant treatment effects on cognition, daily functioning, and global changes. Presently, rating scales for assessment of both behavior and functioning, besides cognition, and evaluation of a patient's global impression are standard outcome measures in AD clinical trials [5]. Also, scales for assessment of advanced AD patients have been developed [6,7], demonstrating the considerably retained abilities of such patients and refuting the belief that patients beyond the moderate stage are not accessible to pharmacological and non-pharmacological treatment. Nevertheless, a need for better instruments, which are more sensitive to disease staging or changes over time, has been acknowledged by the European Medicines Agency [5] and recently confirmed by reviewing the outcome measures used in current clinical trials [8].

Despite the extensive development of rating scales for AD research, the overall assessment of disease progression in routine medical practice remains lengthy and complicated. One reason for that is the need for using a set of scales to assess all AD-relevant symptom domains, which is usually an arduous procedure for both physician and patient/caregiver. Also, most rating scales are not applicable to all AD severity stages, that is, many assessment tools are not consistently sensitive to measure disease progression or therapy effects across the whole patient population.

To discuss the use of current rating scales for evaluation of AD therapy in AD daily medical practice, an expert panel group composed of the authors of this article was set up (14 and 15 September 2007). The panel assumed that an *ideal *AD instrument for daily medical practice should cover all requirements presented in Table 1.

**Table 1:** Characteristics of a multi-domain AD scale for daily medical practice

1) Easy and quick administration by an experienced clinician; about 10 minutes administration time
2) Reliable and valid for AD
3) Covering the AD relevant areas cognition, activities of daily living, behavior, communication and social interaction, and quality of life
4) Applicable to all AD severity stages (with minimal floor and ceiling effects)
5) Useful for monitoring of disease progression in clinical practice
6) Sensitive to measure therapy effects

In this paper, we provide a brief overview on AD scales developed for assessment of cognition, daily function, global impression, behavior, quality of life as well as communication and social interaction, with a focus on their applicability in daily medical practice for monitoring disease progression and therapy effects. The review does not pretend to have included all scales available and used in AD clinical practice so far. It rather summarizes individual scales for the various AD symptom domains and points to the need for a new multi-domain AD scale to enable disease assessment over time in daily medical practice.

## Materials and methods

To discover available AD scales covering the requirements listed in Table 1, we performed a systematic literature search, employing the National Library of Medicines' MEDLINE database. For this purpose, an appropriate search algorithm was established: i) to ensure the inclusion of records about tools used in the assessment of AD (keywords: Alzheimer ('s disease), scale, assessment, rating/rater, questionnaire); ii) to avoid the inclusion of records about measures only for AD diagnosis and staging (keywords: efficacy, outcome, disease progression); iii) to include records on aspects considered neglected in many AD scales (keywords: quality of life, communication, social interaction/activities). Only documents in English, French, German, Italian, and Spanish were considered. Measures specialized on specific topics (for example, assessment of visuospatial functioning, verbal learning) or not developed for AD, dementia, or geriatric patients were omitted. Computerized instruments were not considered due to the technical demands often deemed incompatible with daily practice use. Caregiver burden tools were also excluded since only scales for assessment of AD symptoms were of interest for this literature review.

Two independent reviewers assessed each selected article independently and with regard to its suitability and quality. In cases when the reviewers could determine from the abstract that the selection criteria were not met, the respective articles were rejected; or when a paper could not be rejected with certainty from the abstract, the full text article was obtained and evaluated. In addition, the references included in the selected articles were evaluated to identify further possible documents of interest. Any differences between the reviewers' results were resolved by discussion.

Each rating scale identified from the selected articles was compared with the requirements for a multi-domain AD scale for daily medical practice given in Table 1.

## Results and discussion

Our systematic literature search revealed a total of 1,902 articles published from July 1981 to September 2008. By screening the results, special attention was paid to identification of scales containing items of several AD symptom domains, excluding instruments mainly used for AD diagnosis and staging or screening tests. Reviews, editorials, meta-analyses, or studies published in English, French, German, Italian, and Spanish languages were considered. Most of the selected articles referred to validated and AD-specific tools developed over the last three decades to assess the regulatory relevant AD-symptom complexes: cognition, activities of daily living (ADL), and global changes. The rest of the articles revealed instruments for assessment of behavior, patient's quality of life, and communication and social interaction. Out of all selected articles, 68 relevant AD scales were identified. An overview of the scales grouped by AD symptom domains is provided in Table 2.

**Table 2:** Identified instruments for assessment of AD symptoms

Aspects of AD disorder	Abbreviation	Full name	
Cognitive Impairment	ADAS-Cog	Alzheimer's Disease Assessment Scale - Cognitive Subscale [14]	Rosen *et al*.1984
	BIMC	Blessed Information-Memory-Concentration Test [77]	Blessed *et al*. 1968
	CAMCOG	Cambridge Cognitive Examination [78]	Grevett & O'Brien 2002
	MDRS	Mattis Dementia Rating Scale [79]	Mattis 1976
	MMSE	Mini-Mental State Examination [9]	Folstein *et al*. 1975
	NTB	Neuropsychological Test Battery [17]	Harrison *et al*. 2007
	SCIP	Severe Cognitive Impairment Profile [21]	Peavy *et al*. 1996
	SIB	Severe Impairment Battery [6]	Saxton & Swihart 1989
	SKT	Syndrom-Kurztest [22]	Erzigkeit 1989
Activities of Daily Living	ADL-PI	Activities of Daily Living-Prevention Instrument [31]	Galasko *et al*. 2006
	ADLQ	Activities of Daily Living Questionnaire [80]	Johnson *et al*. 2004
	ADCS-ADL_19/23_	Alzheimer's Disease Cooperative Study Activities of Daily Living Inventory 19- or 23-item Scale [25]	Galasko *et al*. 1997
	B-ADL	Bayer Activities of Daily Living Scale [30]	Hindmarch *et al*. 1998
	Bristol ADL	Bristol Activities of Daily Living Scale [81]	Bucks *et al*. 1996
	BANS-S	Bedfords Alzheimer's Nursing Severity Scale [82]	Bellelli *et al*. 1997
	Barthel ADL	Barthel Activities of Daily Living Index Scale [83]	Mahoney & Barthel 1965
	DAD	Disability Assessment for Dementia [26]	Gelinas *et al*. 1999
	GERRI	Geriatric Evaluation by Relative's Rating Instrument [84]	Schwartz 1983
	IADL	Instrumental Activities of Daily Living Scale [24]	Lawton & Brody 1969
	IDDD	Interview for Deterioration in Daily Living Activities in Dementia [27]	Teunisse *et al*. 1991
	Katz ADL	The Katz Index of Independence in Activities of Daily Living [23]	Katz *et al*. 1963
	PGDRS	Psychogeriatric Dependency Rating Scales [85]	Wilkinson & Graham-White 1980
	RDRS	Rapid Disability Rating Scale [29]	Linn 1976
	PSMS	Physical Self-Maintenance Scale [24]	Lawton & Brody 1969
	SMAF	Functional Autonomy Measurement System [28]	Hebert *et al*. 2001
	Weintraub ADL	Weintraub Activities of Daily Living Scale [86]	Weintraub 1986
Global Impression	ADCS-CGIC	Alzheimers' Disease Cooperative Study - Clinical Global Impression of Change [38]	Schneider *et al*. 1997
	CDR	Clinical Dementia Rating Scale [35]	Hughes *et al*. 1982
	CGI	Clinical Global Impression [34]	Guy 1976
	CIBI	Clinical Interview Based Impression (Parke-Davis) [87]	Knopman *et al*. 1994
	FAST	The Functional Assessment Staging [37]	Reisberg 1988
	GDS	Global Deterioration Scale [36]	Reisberg 1982
	HDR	Hierarchic Dementia Rating Scale [88]	Cole & Dastoor 1983
	NYU CIBIC-Plus	The New York University - Clinician's Interview-Based Impression of Change - Plus Caregiver Input [39]	Reisberg & Ferris 1994
Behavior/Neuropsychology	AMS	Alzheimer's Mood Scale [52]	Tappen & Williams 1999
	IA	Apathy Inventory [49]	Robert *et al*. 2002
	BEAM-D	Behavioral and Emotional Activities Manifested in Dementia [89]	Sihna *et al*. 1992
	BEHAVE-AD	Behavioral Pathology in Alzheimer's Disease Rating Scale [40]	Reisberg *et al*. 1987
	BPRS	Brief Psychiatric Rating Scale [41]	Overall & Gorham 1962
	CERAD-BRSD	Behavior Rating Scale for Dementia of the Consortium to Establish a Registry for Alzheimer's Disease [45]	Tariot *et al*. 1995
	CMAI	Cohen-Mansfield Agitation Inventory [47]	Cohen-Mansfield 1986
	CSDD	Cornell Scale for Depression in Dementia [51]	Alexopoulos *et al*. 1988
	DBD	Dementia Behavior Disturbance Scale [46]	Baumgarten *et al*. 1990
	DMAS	Dementia Mood Assessment Scale [50]	Sunderland *et al*. 1988
	GDS	Geriatric Depression Scale [48]	Yesavage *et al*. 1983
	HoNOS 65+	Health of the Nation Outcome Scale for Elderly People [90]	Burns *et al*. 1999
	NPI	Neuropsychiatric Inventory [42]	Cummings *et al*. 1994
	NRS	Neurobehavioral Rating Scale [91]	Sultzer *et al*. 1995
	RMBPC	Revised Memory and Behavior Problems Checklist [92]	Teri *et al*. 1992
Quality of Life	ADRQL	Alzheimer Disease Related Quality of Life [57]	Rabins *et al*. 1999
	DQoL	Dementia Quality of Life Instrument [58]	Brod *et al*. 1999
	DS-DAT	Discomfort Scale for Dementia of Alzheimer's Type [64]	Hurley *et al*. 1992
	OERS	Observed Emotion Rating Scale [93]	Lawton *et al*. 1999
	PDS	Progressive Deterioration Scale [62]	DeJong *et al*. 1989
	QoL-AD	Quality of Life-Alzheimer's Disease [59,60]	Logsdon *et al*. 1999
	QUALID	Quality of Life in Late-Stage Dementia Scale [61]	Weiner *et al*. 2000
Communication/Social	CLB	Core Linguistic Battery [94]	Bayles *et al*. 1992
Interaction	CPS	Communication Problems Scale [66]	Powell *et al*. 1995
	ERT	Emotion Recognition Test [95]	Shimokawa *et al*. 2000
	MOSES	Multidimensional Observation Scale for Elderly Subjects [96]	Helmes *et al*. 1987
	PKT	Posture Knowledge Test [97]	Mozaz *et al*. 2002
	PPQSA	Partner-Patient Questionnaire for Shared Activities [98]	Reilly 2006
	SCS	Social Competence Scale [99]	Zigler & Phillips 1961
Multi-domain	BGP	Behavioral Rating Scale for Geriatric Patients [71]	van der Kam *et al*. 1971
	GBS	Gottfries-Brane-Steen Scale [68,69]	Gottfries *et al*. 1982
	NOSGER	Nurses' Observation Scale for Geriatric Patients[70]	Brunner & Spiegel 1990
	SCAG	Sandoz Clinical Assessment Geriatric Scale [67]	Shader *et al*. 1974
	VL	The Vienna List [72]	Porzsolt *et al*. 2004

### Cognition

A total of nine instruments for assessment of cognitive impairment were identified by the literature search (Table 2). They comprise measures of cognition in patients with mild to severe AD, which have been used in different clinical settings. The most often used scale for assessment of cognition is the Mini-Mental State Examination (MMSE). It was designed as a practical tool for grading the cognitive state of patients [9]. The target population is patients with cognitive disturbances derived from dementia syndromes, affective or personality disorders. The scale comprises 11 questions or simple tasks concerning orientation, memory, attention and language to evaluate the patient's cognitive state. Other mental or behavioral aspects are excluded, thus it requires only 5 to 10 minutes for a trained rater to administer it. The MMSE is the standard staging and assessment tool in AD. Given the widespread use of the MMSE, clinical and research findings can be easily compared. However, there are several drawbacks limiting the utility of MMSE. Some items are judged to be relatively easy and therefore patients with mild AD are not sensitively evaluated due to ceiling effects [10]. In contrast, floor effects limit the application to patients with more advanced AD. Furthermore, using MMSE for staging of AD severity neglects other more patient- and caregiver-relevant domains of AD assessment, such as function and behavior. Also, measurement error, practice effect, or other factors such as age, education or cultural background may impair the sensitivity and validity of the assessment of disease progression and pharmacological treatment effects [11-13].

Another scale used in almost all clinical trials on symptomatic AD therapy is the Alzheimer's Disease Assessment Scale (ADAS). It was designed to assess both cognitive and non-cognitive AD-specific symptoms [14]. The cognitive subscale, ADAS-Cog, is a standard tool in pivotal clinical trials to detect therapeutic efficacy in cognition. It consists of 11 subtests related to memory, praxis, and language. The non-cognitive subscale of ADAS comprises 10 items evaluating mood and behavioral changes. Depending on the AD severity stage of a patient, the administration of ADAS-Cog takes 30 to 45 minutes. In contrast to the ADAS-Cog, the non-cognitive subscale is rarely used. The ADAS-Cog appears to be most adequate for patients with moderate AD. Patients with mild cognitive impairment and mild AD are subject to ceiling effects and patients in advanced AD stage are subject to floor effects, in part due to eroding language abilities. In general, the measurement error of ADAS-Cog limits disease progression monitoring, especially in short-term evaluations [15]. As the original ADAS-Cog neglects some cognitive functions, such as planning and executive function, several additional subtests have further been developed [16].

As the standard cognitive tools MMSE and ADAS-Cog are not considered sensitive enough to measure treatment effects in early disease stages optimally, a Neuropsychological Test Battery (NTB) has recently been developed to fill this gap [17]. The NTB is designed to emphasize the assessment of memory and executive function in patients with mild to moderate AD, combining nine previously validated cognitive tests. In addition, it provides an index of global cognitive function in mild AD patients. The completion of the NTB takes about 40 minutes.

To enable the assessment of cognition in later AD stages, the Severe Impairment Battery (SIB) was developed [6,18]. The SIB, rather than rating erroneous performance, relies on the appraisal of preserved abilities in nine cognitive domains: social interaction, memory, orientation, language, attention, praxis, visuospatial abilities, constructional abilities and orientation to name. A maximum of 30 minutes is required for administration. A short SIB version [19] and a version based on the SIB-language domain, SIB-L [20], have also been developed. Another brief, reliable and valid measure of cognitive function in severely demented AD patients is the Severe Cognitive Impairment Rating Scale [21]. For staging the severity of cognitive deficits and assessing the benefits of AD therapy, the Syndrom-Kurztest [22] has extensively been used in earlier clinical trials. It is well accepted by patients, hospital clinicians, and general practitioners due to its brevity and simplicity.

### Activities of daily living (ADL)

Among numerous ADL scales available for several decades to rate the degree of disability or the need for assistance in geriatric population, 17 ADL instruments were identified by the literature search presented here (Table 2). In general, basic ADL, including self-maintenance skills such as walking, feeding, and dressing, are distinguished from the instrumental activities of daily living (IADL), addressing more complex activities such as shopping, cooking, handling finances, or using the telephone or transportation. The oldest and most widely used tools to assess functioning are the Katz Index of ADL, which covers six basic ADL (bathing, dressing, toileting, transfer, continence, feeding) [23], and the ADL/IADL scales developed by Lawton and Brody (1969), which contain both basic ADL and IADL [24].

The Alzheimer's Disease Cooperative Study (ADCS) tested 45 ADL items for use in AD clinical trials and found 27 to be widely applicable for assessment of functional capacity across a wide spectrum of severity [25]. The 19-item version (ADCS-ADL_19_), covering mainly basic ADL, is used for the assessment of patients with more severe AD, while the 23-item version (ADCS-ADL_23_) includes more complex ADL for the assessment of mild to moderate AD, such as reading books or magazines, pastime activities, or household chores. Ratings take about 20 minutes and are based on information obtained from the patient and caregiver. The scores range from 0 to 78, higher scores indicating less functional impairment. Another AD-specific and commonly used scale for functional assessment is the Disability Assessment for Dementia [26]. Based on an interview with the caregiver, the clinician evaluates whether the patient needs support concerning initiation, organization and planning, and effective performance in 10 areas of functioning, including basic, instrumental, and leisure daily activities. Other functional instruments used in different clinical settings for assessment of AD patients are the Interview for Deterioration in Daily Living Activities in Dementia [27], the Functional Autonomy Measurement System [28], and the Rapid Disability Rating Scale [29]. The Bayer Activities of Daily Living Scale (B-ADL) was developed for patients with mild cognitive impairment or mild to moderate dementia to measure deficits in the performance of everyday activities [30]. It is a useful tool for evaluation of treatment effects and the progress of dementia in general practice primary care. Other functional instruments particularly used for patients with mild cognitive impairment are the Activities of Daily Living-Prevention Instrument [31], developed to rate ADL in prevention of dementia studies, and the Functional Assessment Questionnaire [32]. A recent review of the IADL scales has pointed to a need for an improvement in the psychometric properties of the currently available IADL instruments in order to justify better their usefulness in clinical practice [33].

### Global impression

The concept of Clinical Global Impression (CGI) for evaluation of pharmacological treatment effects was introduced by Guy, 1976 [34]. The CGI scales rely on the clinician's rating of global severity and/or change in patient's clinical condition. Global measures developed for use in dementia are normally based on a comprehensive and (semi-) structured interview with the patient and other suitable (caregiver-) informants [13]. The scales characterize clinically manifested changes of dementia, based on a multidimensional evaluation of cognitive, functional, and behavioral symptoms. The global measures selected by our literature search (Table 2) include global severity scales, such as the Clinical Dementia Rating [35], the Global Deterioration Scale [36], and the Functional Assessment Staging [37], and global change scales, such as the Alzheimer's Disease Cooperative Study - Clinical Global Impression of Change [38] and the New York University - Clinician's Interview Based Impression of Change - Plus Caregiver Input [39]. The administration time of global assessment scales ranges from 5 to 45 minutes depending on the amount of information to be gathered. The main advantage of global measures is that they take into account multiple domains of real life information and, in contrast to performance-based tools such as MMSE, a detected change in the patient's condition is clinically meaningful, as an experienced clinician can detect the change. On the other hand, most global measures do not allow separate monitoring of changes over time in several AD-relevant domains.

### Behavior

During the last decades, several instruments have been developed to assess behavioral and psychological symptoms in AD patients. Our literature search identified 15 scales, including instruments for assessment of behavior and mood. The BEHAVE-AD was developed to assess behavior in patients with AD apart of cognitive symptomatology [40]. It covers symptoms in seven categories: paranoid and delusional ideation, hallucinations, activity disturbances, diurnal rhythm disturbances, aggressiveness, affective disorders and anxieties, and phobias. The administration time is about 20 minutes, and behavior is rated as mild, moderate, or severe. An early developed instrument for assessment of behavior, which is still often used in clinical practice and psychopharmacological research, is the Brief Psychiatric Rating Scale (BPRS). It contains 18 items grouped in five factors - depression, agitation, cognitive dysfunction, hostile suspiciousness, and psychotic distortion - rated on a seven-point severity scale [41]. The administration of the BPRS takes 20 minutes by an experienced clinician and is based on observation of and self-reporting by the patient. Presently, the most widely used behavior scale in AD is the Neuropsychiatric Inventory (NPI). It was developed to compensate for shortcomings of the BEHAVE-AD, which neglects symptoms related to apathy, irritability, or disinhibition [42]. The NPI distinguishes between frequency and severity of symptoms; it is informant-based and requires about 30 minutes for administration. A brief version was developed, which relies on a questionnaire and assesses only symptom severity [43]. However, as the NPI contains contradicting opposing symptoms (euphoria and depression), the value of the NPI total score has recently been called into question [44] and it seems more important to look at the scoring of each behavioral domain.

Other instruments used in clinical settings for rating behavioral abnormalities in patients with AD are the Behavior Rating Scale for Dementia of the Consortium to Establish a Registry for Alzheimer's Disease [45], the Dementia Behavior Disturbance Scale [46], as well as instruments targeting specific behaviors, such as the Cohen-Mansfield Agitation Inventory [47] for agitation and aggressiveness, the Geriatric Depression Scale [48] for depression, and the Apathy Inventory [49]. In addition, there are instruments designed specifically for assessment of depression severity and mood changes in demented patients, such as the Dementia Mood Assessment Scale [50], the Cornell Scale for Depression in Dementia [51], and the Alzheimer's Mood Scale [52].

### Quality of life

In the second half of the 1990 s, following a general medical trend of moving away from mainly symptom treatment towards patient-centered medical care, the development of scales for assessment of quality of life (QoL) became a new challenge. Most dementia QoL-scales refer to Lawton's model of QoL in dementia [53]. According to this model, QoL is the result of a dynamic interaction between four patient-relevant dimensions: psychological well being, perceived quality of life, behavior competence, and environment [54]. QoL scales contain aspects not considered in most conventional scales, such as interpersonal relationships, self-esteem, living environment, being useful, giving meaning to life, or financial situation. In general, QoL instruments are specific to the severity stage of AD and differ with regard to the domains covered. Most QoL scales are short and easy to administer. However, there has been substantial debate whether patients with AD, especially in more advanced stages, can reliably report on their QoL and whether caregiver reports are an appropriate alternative [55]. Furthermore, the individual designation of QoL is rather flexible and inherently differing among people, which makes the common understanding of what is important for the QoL of a person with dementia a challenge for researchers and clinicians. To rate the extent to which treatment goals individually defined by the patient, caregiver or physicians are achieved, the method of goal attainment scaling was developed and applied to both geriatric and dementia populations [56].

Among the QoL scales selected in our search were the Alzheimer Disease Related Quality of Life (ADRQL) [57], the Dementia Quality of Life Instrument (DQoL) [58], the Quality of Life - Alzheimer's Disease (QoL-AD) [59,60], and the Quality of Life in Late-Stage Dementia Scale (QUALID) [61]. The ADRQL is an observer-rated instrument, which measures positive and negative behavior across five domains: social interaction, awareness of self, feelings and mood, enjoyment of activities, and response to surroundings. It is appropriate to use in daytime activities settings and is applicable to all stages of dementia. The DQoL instrument is a 29-item scale designed for assessment of QoL in mild to moderate demented patients. Ratings are based on an interview with the patients and give estimates for positive and negative affects, feelings of belonging, sense of aesthetics, and self-esteem. The QoL-AD was developed for both patients and caregivers to measure QoL in AD, and can be used for patients with MMSE score > 10. It includes 13 items providing assessment of mood, physical health, memory, relationship, self-esteem, and current situation. Another QoL instrument specifically designed for AD patients is the Progressive Deterioration Scale [62]. It is relatively easy to administer, only 10 to 15 minutes to complete, and has been often used in clinical settings. In addition, the QoL measures include the Schedule for the Evaluation of Individual Quality of Life - Direct Weighing (SEIQoL-DW), a self-rating instrument which allows patients to designate and weigh the most important domains affecting their quality of life [63]. Another easy to use but requiring extensive training for administration instrument is the Discomfort Scale - Dementia of Alzheimer Type [64]. It is an observation assessment scale developed to measure discomfort in the elderly with AD.

### Communication and social interaction

Improving QoL of AD patients requires specific instruments to assess problems related with the ability of patients to communicate and interact with others. Communication breakdown in AD results from disturbances in semantic and linguistic processes or memory deficits and causes a large number of symptoms. The diminished ability to communicate leads to a decline in the quality of social interactions and increased caregiver burden [65]. Our literature search revealed seven instruments for assessment of symptoms of communication breakdown. An example for evaluation of semantic and pragmatic problems in communication with AD patients is the Communication Problems Scale [66]. It is a 16-item inventory administered to caregivers, who estimate the frequency of each communication behavior on a five-point score.

### Selected multi-domain scales

In this study, the literature survey was based on pre-defined requirements for a multi-domain AD scale for therapy evaluation in daily medical practice in order to identify existing AD scales which might cover the criteria, respectively. Most of the selected scales (Table 2) appear to evaluate aspects which can be predominantly assigned to a single AD symptom domain, namely cognition, ADL, or behavior, including also a few scales selected as tools for assessment of patients' well-being, affects, apathy, mood, pain, and communication/social interaction. A number of global and QoL scales were also identified by the search. In general, conventional AD symptom domains can not be allocated to QoL scales. These assessment tools are inherently multidimensional, delivering a subjective patient perspective; however, they can not replace the detailed evaluation of function or behavior.

Besides these scales, our literature survey yielded only a few scales that allow assessment of several AD-domains (cognition, ADL, behavior, including also communication/social interaction) and cover at least some of the requirements for a multi-domain instrument for therapy evaluation in daily medical practice, listed in Table 1. The selected multi-domain scales are: the Sandoz Clinical Assessment Geriatric (SCAG) [67], the Gottfries-Brane-Steen (GBS) [68,69], the Nurses' Observation Scale for Geriatric Patients (NOSGER) [70], the Behavioral Rating Scale for Geriatric Patients (BGP) [71], and the Vienna List [72]. Table 3 provides a brief overview on the AD-domains covered by the scales, the patient population, scale administration and application.

**Table 3:** Brief overview of the selected multi-domain AD scales

Scale	Domains/dimensions (number of items)	Patient population	Rating	Rater	Item scoring	Application
BGP (van der Kam *et al*. 1971)	Caregiver dependency (9), Social skills (7), Aggressiveness (5), Psychoorganic syndrome (11), Depressiveness (3)	Moderate to severe AD	Observation	Nurse, Caregiver	3-point	Used in clinical trials for assessment of behavior in psychogeriatric patients; the BGP-care dependency subscale reflects cognitive and functional characteristics related with increased need for care
GBS (Gottfries *et al*. 1982)	Intellect (11), Emotion (3), Self-care (6), Behavior (6)	Mild to severe AD	Observation/interview	Physician, Psychologist, Nurse	7-point	Global assessment tool for evaluating dementia symptoms; it is not suitable for diagnostic purposes but for repeated measurements of patients participating in clinical trials for assessment of drug treatment effects
NOSGER (Brunner & Spiegel 1990)	Memory (5), IADL (5), Self-care (5), Mood (5), Social behavior (5), Disturbing behavior (5)	Mild to moderate AD	Observation	Nurse, Caregiver	5-point	Developed for longitudinal studies in psychogeriatrics; used in several European and North American centers; suitable for dementia screening
SCAG (Shader *et al*. 1974)	Cognitive dysfunction (4), Interpersonal relationship (4), Apathy (4), Affect (3), Somatic function (3)	Senile dementia	Observation/interview	Clinician	7-point	General-purpose rating scale developed for evaluation of pharmacotherapy in senile dementia; widely used in drug research trials during 1980's
VL (Porzsolt *et al*. 2004)	Communication (15), Negative affect (10), Bodily contact (5), Aggression (4), Mobility (6)	Severe AD	Observation	Physician, Nurse	7-point	A relatively new developed proxy-rating QoL instrument for documentation of the outcome of geriatric inpatient rehabilitation

AD, Alzheimer's disease; BGP, Behavioral Rating Scale for Geriatric Patients; GBS, Gottfries-Brane-Steen; NOSGER, Nurses' Observation Scale for Geriatric Patients; SCAG, Sandoz Clinical Assessment Geriatric; VL, the Vienna List

The SCAG is a general-purpose rating scale to assess changes following treatment. It contains 18 items, comprising the assessment of agitation, cognitive dysfunction, depressed mood, and withdrawal, scored on a seven-point scale. An additional item, item 19 of the SCAG, is included for global severity rating. A skilled clinician administers the scale over 15 to 30 minutes. Rating is based on observed behavior and not on the patient's own impression. The SCAG was designed specifically for evaluation of pharmacotherapy in senile dementia and for a time was widely used as an outcome measure in drug research [73].

The GBS was designed to measure the degree of dementia and to profile dementia syndromes. It consists of 26 items divided into four subscales measuring motor performance, intellectual disturbances, and emotional impairment; the fourth subscale, entitled self-care, estimates different dementia symptoms' characteristics over time, comprising the assessment of confusion, irritability, agony, anxiety, mood, and restlessness. Only trained clinicians (physicians, psychologists and nurses) can administer it. The rating takes about 30 minutes and is based on observation of the patient during a semi-structured interview. The scale is not meant for diagnostic purposes but for repeated measurements of patients participating in clinical trials and evaluation of drug treatment effects.

The NOSGER is a rating scale that covers a wide range of behavioral pathology relevant to daily functioning and independent of gender or social status of the patients [70]. It consists of 30 items which measure impairment in the following areas: memory, IADL, basic ADL, mood, socialization, and disturbing behavior. Each item is rated on a five-point scale according to frequency of occurrence. Rating takes about 20 minutes and is based on direct observation of daily behavior by the nurse/caregiver over a two-week period. The scale was developed for rating the frequency of behavioral disturbances, but appears suitable for dementia screening as well. Validation studies have shown good acceptance of the scale, high inter-rater and test-retest reliability, and high correlations of NOSGER dimension scores with results of other scales [70].

Distinct from the first three multi-domain scales, the BGP is predominantly a scale for behavioral assessment of geriatric patients. It has been used in Europe since 1971 and has demonstrated good reliability in measuring longitudinal changes. Thirty-five items are included in the BGP, covering aspects of cognition, function, and behavior. The BGP-Care dependency subscale (nine items) is particularly used for assessment of cognitive and functional characteristics associated with increased need for care. The rating is based on observed behavior for a week by the clinical nursing staff. The scores range from 0 to 70, higher scores reflecting increased severity.

In contrast to the BGP, the Vienna List is a relatively new instrument. It has been developed as a proxy-rating measurement for QoL in patients with severe dementia [72]. The scale consists of 40 items grouped into five factors describing behavior of demented patients. The scale has also been validated as a useful, differentiating, and practical tool for documentation of the outcome of geriatric inpatient rehabilitation [74].

Each of the identified five multi-domain scales was compared with the requirements listed in Table 1 to reveal its relevance to daily medical practice. It shows that only one of the selected scales, the GBS, is applicable to all AD severity stages (mild to severe). The SCAG can also differentiate between four groups of individuals: healthy, minimum dementia, depression, and severe dementia. However, SCAG was developed for evaluation of the most common clinical manifestations of geriatric dysfunction and is not specific to problem behaviors common among patients with AD. The NOSGER is applicable only for mild to moderate AD patients, whereas the BGP and the Vienna List are designed for moderate to severe and severely demented patients, respectively.

All the scales were reported as easily administrable and practical for use, requiring a relatively short time for administration. However, both the BGP and the Vienna List appear a bit lengthy (35 and 40 items, respectively). For comparison, the patient and caregiver versions of the 13-item QoL-AD scale can be completed on average in 10 minutes [59]. Two of the scales, the NOSGER and the BGP, are based on observations taken by nurse/caregiver, whereas the other three scales are based on observations by a trained clinician. Validation data (inter-rater, test-retest, construct and concurrent reliability) are published for all selected scales. Most of the scales have shown a good sensitivity to measure changes with disease progression. The SCAG and GBS have also proven their reliability in monitoring treatment effects over time.

A common disadvantage of all five scales is the emphasis given to the assessment of a single AD-symptom domain despite the scales' multi-domain structure. Particularly, the assessment of behavior is prevailing in the NOSGER, BGP, and the Vienna List, whereas the cognitive evaluation is central to the GBS and SCAG scales.

The GBS is applicable to all AD severity stages but its disadvantage is the inclusion of only basic ADL, which can lead to ceiling effects in mild AD patients. Also, no IADL are scored and QoL is not assessed. Furthermore, the GBS contains only one language item (*language disturbances*) while the NOSGER, BGP, and the Vienna List, which were developed on a more caregiver-based perspective, also take into account aspects related to social interaction. For example, the NOSGER contains items such as "is interested in what is going on around him/her", "helps others as far as physically able", or "when asked questions, seems quarrelsome and irritable". Unfortunately, these scales have limitations concerning the severity AD stage to which they can be applied.

### Development of a new multi-domain AD scale

Despite the variety of validated AD-scales revealed by our literature survey, none of the selected instruments fulfilled all criteria for a multi-domain AD scale given in Table 1. The development of a new instrument that can address multiple domains at all stages of AD severity while remaining sensitive to changes and therapy effects seems to be an ambitious goal. To achieve it, the approach of combining available AD scales appears to be inappropriate as these scales have different designs and scopes of assessment. Hence, we believe that the new multi-domain instrument should be developed with a novel design that may best serve primary care physicians to assess severity of AD symptoms, disease progression, and therapy effects in daily medical practice. How should such a scale be created and used in practice? First of all, practice-relevant endpoints that cover a broad spectrum of AD symptoms and enable global clinical evaluation of the disease progression should be selected and included in an appropriate test-frame applicable to all disease severity stages. We believe that an external assessment carried out by a medical practitioner and based on an interview with the patient and/or patient's caregiver would be the most appropriate format for the new practical instrument. Given the broad disease severity range of the scale, special attention should be given to eventual floor and ceiling effects, which can compromise the measurement. Ceiling effects may be expected for patients in the early AD stage; therefore, we would propose developing the scale only for assessment of patients with clearly established diagnosis of AD and exclude prodromal stages of dementia, such as mild cognitive impairment. Another challenge is the development of a test-frame applicable to a broad scope of AD patients differing in terms of cultural background. This may be achieved by using a scale allowing adjustments to individual patients without loosing sensitivity and reliability. An interview based on a given example of event or action, that is, scenario, for assessment of AD symptoms would be an appropriate test-format; thus, alternative scenarios could be adapted to the individual patient. Validated translations in many languages would also be required. Besides being a reliable and sensitive tool, such a scale should also be user-friendly to become often used in daily clinical practice. We assume the new scale for clinical practice should take about 15 to 30 minutes for administration by an experienced clinician, who is supposed to be familiar with the background clinical information of the patient. At following assessments, such a scale would enable the user to obtain and evaluate data faster and at ease. For accurate assessment, the clinician should use a broad source of information about the patient, for example, from a caregiver, a relative, or the patient himself. Accordingly, an informant interview would be an appropriate form of administration.

Altogether, the development of the new instrument would require considerable effort, combining the knowledge and experience of primary care clinicians, researchers, and caregivers from different countries. As a first step towards scale development, a panel meeting with medical and caregiver experts with long-term experience in AD therapy was organized to discuss the content of this new scale and to establish a strategy for scale development. In general, a consensus was achieved that most AD scales commonly used in clinical practice do not comprise all requirements for a practical multi-domain AD scale. Most are standardized assessment tools to measure predominantly the severity of major dysfunction in a particular AD domain, but are not sufficiently sensitive to all AD stages, therefore are not easily applicable for tracking changes in routine medical practice and comparable in different patient populations. Moreover, several aspects of cognition and communication/social interaction, which are of great impact for daily medical practice, appear to be neglected and/or not well captured by the commonly used AD scales. Cognitive aspects such as misidentification, learning aptitude, self-disclosure, decision making, self-determination, and disturbed/slowed response to external stimuli, seem rarely presented in the selected standard measures. The following aspects of communication and social interaction were also considered seldom presented in AD scales: responsiveness, correct use and interpretation of gestures and facial expressions, implementation of commands, cooperation in care and daily living, social involvement, adaptability, relationships with family/friends, and intimacy of contacts (informal/formal).

The extensive discussion on these specific practical challenges contributed to the primary selection of practice-relevant assessment criteria to be included in the new scale. In total, 16 items with high clinical relevance were selected for the new instrument and grouped as follows: cognition (three items), communication (three items), behavior (five items), and activity of daily living (ADL) (three items). Two additional endpoints for global clinical evaluation of patients' quality of life and caregiver burden were considered relevant as well. As a next step, a framework appropriate for assessment of patients at all AD severity stages was developed based on stage-specific scenarios (early, middle, and late) and relevant examples for assessment of each scale item. For an individual patient, the scenario should be chosen by the clinician as dependent on the clinician's global impression of patient's AD-relevant symptoms and their severity. Such a test framework enables also the use of patient-adapted scenarios, which makes it appropriate for assessment of patients with diverse cultural, social, or educational backgrounds. Further considerations regarding test utility were discussed at the panel meetings, leading to the final set up of a clinical trial design whose primary goal was to evaluate the validity, reliability, and sensitivity of the new instrument. This clinical trial has recently been performed, demonstrating the validity and reliability of the new instrument. Some of the clinical trial results have recently been presented at the 11th International Geneva/Springfield Symposium on Advances in Alzheimer Therapy [75,76] and will be published in a separate manuscript currently in preparation.

## Conclusions

Given the literature review results and extensive discussions at the expert panel meetings, a new AD scale designed for multidimensional assessment of symptoms in daily medical practice and applicable to all AD severity stages is definitely needed. Such a scale would serve experienced clinicians and researchers in monitoring patient-relevant AD symptoms over time in clinical practice, and in evaluating the efficacy of new treatments under development. A great advantage of such a rating tool for clinical practice is the possibility to easily profile AD-relevant symptoms for a patient, and thus get a broad overview of a patient's disease status and therapy effects over time. Also, such a tool may be used as a sensitive global measure of changes across severity stages, and thus provide evaluations of therapy effects in long-term assessments to caregivers or reimbursement agencies.

## Abbreviations

AD: Alzheimer's disease; ADAS-Cog: Alzheimer's Disease Assessment Scale - Cognitive Subscale; ADCS-ADL_19/23_: Alzheimer's Disease Cooperative Study Activities of Daily Living Inventory 19- or 23-item Scale; ADL: activities of daily living; ADRQL: Alzheimer Disease Related Quality of Life; B-ADL: Bayer Activities of Daily Living Scale; BEHAVE-AD: Behavioral Pathology in Alzheimer's Disease Rating Scale; BGP: Behavioral Rating Scale for Geriatric Patients; BPRS: Brief Psychiatric Rating Scale; CGI: Clinical Global Impression; DQoL: Dementia Quality of Life Instrument; GBS: Gottfries-Brane-Steen; IADL: instrumental activities of daily living; MMSE: Mini-Mental State Examination; NOSGER: Nurses' Observation Scale for Geriatric Patients; NPI: Neuropsychiatric Inventory; NTB: Neuropsychological Test Battery; QoL: quality of life; QoL-AD: Quality of Life-Alzheimer's Disease; QUALID: Quality of Life in Late-Stage Dementia Scale; SCAG: Sandoz Clinical Assessment Geriatric; SEIQoL-DW: Evaluation of Individual Quality of Life - Direct Weighing; SIB: Severe Impairment Battery; SIB-L: Severe Impairment Battery - Language; VL: The Vienna List.

## Competing interests

PR, SF, SG, RI, and BW are members of an Advisory Board for Alzheimer's disease coordinated from Pierrel Research Europe (Essen, Germany), for which they received an honorarium from Merz Pharmaceuticals (Frankfurt, Germany). FT is an employee of Merz Pharmaceuticals and receives a fixed salary. SG, RI, BW and PR are also members of the Advisory Board at Lundbeck, in which capacity they have received consulting fees. In the last five years, PR has received honoraria or royalties from Eisai, Wyeth, Novartis, Janssen, and GE Healthcare. RI has received consultant fees from APK, Austroplant, BDI, Beltz Test, BOD, Caritas Siegen, Double Helix Development, Eisai, Friedrichverlag, GE Healthcare, Hogrefe, IFE, Janssen, KDA, Landesinitiative Demenz Service NRW, LVR Düren, Medical Tribune, Med. Komm., Novartis, Pfizer, Pfrimmer Nutritia, Pierrel, Schwabe, Thieme, Urban & Vogel, and Westermayer.

## Authors' contributions

All of the authors contributed to the study conception and selection of criteria for the design of a new assessment instrument. FT coordinated the literature survey and drafted the manuscript. PR, RI, SG, SF and BW provided critical remarks on drafts and guided the manuscript through its development. All authors read and approved the final manuscript.
